# The Function of *RcAG2* and *RcFUL* in the Flower Shape Change of *Rosa chinensis* ‘Viridiflora’

**DOI:** 10.3390/plants15010011

**Published:** 2025-12-19

**Authors:** Jinfeng Zhang, Hui Liao, Yipeng Yang, Xixi Zhang, Caijie Yi, Lina Song, Zijing Li, Hua Zhang, Peng Ji

**Affiliations:** 1 College of Horticulture, Heilongjiang Bayi Agricultural University, Daqing 163318, China; zhang20231632023@163.com; 2 Institute of Botany, Beijing Academy of Forestry and Landscape Architecture, Chaoyang District, Beijing 100102, China; 15600791426@163.com (H.L.); yyp18832076906@163.com (Y.Y.); petunia@126.com (X.Z.); yi2zhao@163.com (C.Y.); songln_009@126.com (L.S.); lizijing8341@126.com (Z.L.)

**Keywords:** *Rosa chinensis* ‘Viridiflora’, flower type, flower development, *RcAG2*, *RcFUL*

## Abstract

The floral morphology of *Rosa chinensis* significantly influences its ornamental value. However, the molecular mechanisms underlying specific floral types remain poorly understood. Viridiflora, a stable genetic variant of *R. chinensis*, exhibits homeotic transformation of floral organs into sepal-like structures, providing a valuable model for studying floral organ identity and development. In this study, Viridiflora was compared with Old Blush to elucidate floral development through morphological observation, transcriptomic profiling, and functional genetics. Four distinct developmental stages were defined, encompassing the formation of sepal, petal, stamen, and pistil primordia. Transcriptome analysis identified candidate genes associated with the Viridiflora phenotype, among which *RcAGAMOUS2* (*RcAG2*) and *RcFRUITFULL* (*RcFUL*) were selected for in-depth functional characterization. The proteins encoded by these two genes are hydrophilic, lack signal peptides and transmembrane domains, and contain multiple phosphorylation sites. They feature typical MADS-box family domains and show close phylogenetic affinity to *Rosa rugosa*. Subcellular localization showed their nuclear presence. Heterologous overexpression of *RcAG2* and *RcFUL* in Arabidopsis resulted in notable phenotypic alterations: *RcAG2* caused petal reduction and stamen exposure, while *RcFUL* led to greenish, leaf-like petals with pigmentation gradients, increased sepal number, and failed seed set. Conclusion: These results suggest that *RcAG2* and *RcFUL* play key roles in floral organ development through genetic regulation, providing a theoretical foundation for further research on floral development in *R. chinensis.*

## 1. Introduction

*Rosa chinensis*, a semi-evergreen shrub or climbing plant of the genus *Rosa* (Rosaceae), is revered as the “queen of flowers” and ranks among China’s top ten famous flowers. It is also one of the world’s most widely cultivated ornamental plants, prized for its diverse flower forms, fragrances, and colors [1]. While current research on *R. chinensis* flower morphology has largely focused on petal number, less attention has been given to the floral-organ variant cultivar *Rosa chinensis* ‘Viridiflora’ (hereafter Viridiflora). In Viridiflora, petals, stamens, and pistils are transformed into sepal-like structures, resulting in entirely green flowers. This unique morphology and coloration make it highly valued in ornamental horticulture and appreciated in international markets. *Rosa chinensis* ‘Old Blush’ (hereafter Old Blush), a recurrent-flowering ancient Chinese rose with high adaptability, is a key genetic resource in breeding and a major ancestor of modern roses. Viridiflora, a natural mutant of ancient Chinese roses, is closely related to Old Blush [1,2]. Due to its genetic stability and representative traits, Old Blush is frequently used as a control in rose developmental studies and has been proposed as a model cultivar [3,4].

Flower structure consists of a peduncle—an extension of the branch—topped by a receptacle from which sepals, petals, stamens, and carpels arise as modified leaves, illustrating the developmental plasticity of floral organs [5]. In some species, sepals are often mistaken for petals due to their size and color, as seen in *Hydrangea macrophylla* [6], *Clematis florida* [7], and *Aquilegia viridiflora* [8]. In other plants, such as *R. chinensis* [9], *Paeonia lactiflora* [10], *Camellia japonica* [11], stamen or pistil conversion into petals underlies the formation of double flowers. Unusual petal morphologies—such as slender or curled forms—also contribute to floral diversity in species like *Chrysanthemum morifolium* [12] and *Phalaenopsis aphrodite* [13]. The ABC model of floral organ identity, proposed by Coen and Meyerowitz in 1991 based on studies in *Arabidopsis thaliana* and *Antirrhinum majus* describes how combinatorial gene functions specify sepal (A-class), petal (A + B), stamen (B + C), and carpel (C-class) development [1,14]. However, ectopic expression of ABC genes outside floral whorls typically fails to convert leaves into floral organs [15,16]. This model was later expanded to the “ABCDE model”, incorporating D-class genes essential for ovule development and E-class genes (*SEPALLATA*) required for the identity of all floral organs [17,18,19]. Mutations or misregulation of these genes often lead to homeotic transformations. Notably, co-expression of the A, B, C, and E-class genes can convert leaves into floral organs in *Arabidopsis* [18,20].

Abnormal floral phenotypes in *R. chinensis* are generally attributed to mutations or altered expression of floral organ identity genes, often accompanied by changes in phytohormone signaling. Northern blot analyses revealed that Viridiflora and *Rosa hybrid* ‘Motrea’ (hereafter Motrea) exhibit suppressed expression of *AGAMOUS* (*AG*), a C-class gene essential for stamen and carpel formation, which also influences meristem activity and gibberellin biosynthesis [21]. *RcAG* is expressed throughout bud development in normal roses but is silent in early bud stages in both Viridiflora and Motrea [22]. Furthermore, expression levels of *RcAG* are significantly lower in Viridiflora compared to Old Blush, likely due to methylation at four CpG sites within its promoter region [23]. Similar phenotypes occur in *Thalictrum thalictroides*, where *ThtAG1* suppression leads to stamen and pistil conversion into sepals [24]. In addition, *RcAG* is mainly expressed in the third and fourth whorls of rose flower and has the function of terminating the development process. Suppression of *RcAG* expression could lead to increase the number of rose petals [25]. The MADS-box transcription factor *FRUITFULL* (*FUL*) belongs to the *APETALA1/FRUITFULL* (*AP1/FUL*) subfamily, which includes three evolutionary lineages: *euAP1*, *euFUL*, and *Ful-like*. Among them, euFUL and FUL-like proteins are functionally redundant and have conserved C-terminal motifs (L/MPPWML) [26,27]. In *A. thaliana*, *AtFUL* plays a role in regulating leaf, flower, and fruit development. Transient silencing *RcFUL* in the rose could lead to a delay in flowering time, indicating that this gene was a positive regulatory factor for flowering [28].

*RcAG* and *RcFUL* play significant roles in the organ development of flowers, but there are differences in their expression and function. At present, *RcAG* is mostly documented in studies investigating double-flowered roses, affecting the termination of the second whorl of flower organs. *RcFUL* is mostly related to the flowering time of roses and the development of carpels. Comparing MIKC^C^-type MADS-box gene expression between Viridiflora and Old Blush, revealing up-regulation of *RcFUL2*, *RcFUL3*, and *RcFUL5* during carpel differentiation in Viridiflora, absence of *RcAP3.3* expression in early developmental stages, and organ-specific expression of *RcAP1.2*, *RcAG2*, and *RcSEP4.3* exclusively in Viridiflora leaves [1]. Studies have shown that At*FUL* can have a direct or indirect impact on miRNA172 [29,30]. Small RNA sequencing identified significant down-regulation of miR172 in Viridiflora petals, stamens, and pistils, coupled with up-regulation of its target *RcAP2*, suggesting miR172 may negatively regulate *RcAP2* to promote phyllody [31,32]. In addition, it was found that At*FUL* can directly bind to the promoter of *AP2* expressed in inflorescence meristems and pistil tissues [33].

To further investigate the molecular mechanisms underlying the unique floral morphology of Viridiflora, this study combined microscopic observation of floral bud development with transcriptome analysis to identify candidate genes. Functional characterization via subcellular localization and heterologous overexpression in *A. thaliana* was performed to elucidate their roles in floral organ development. These findings provide new insights into the genetic regulation of ornamental traits in *R. chinensis* and establish a theoretical foundation for targeted breeding strategies.

## 2. Results

### 2.1. Morphological Dynamics of Floral Bud Differentiation in Viridiflora and Old Blush Roses

**Figure 1:**
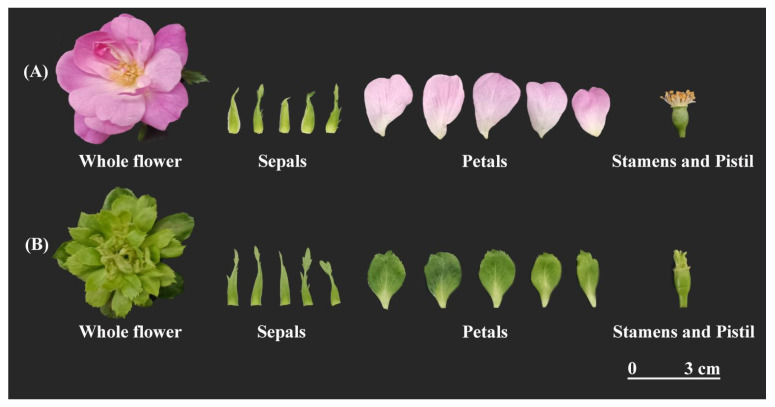
Floral organ display of *Rosa chinensis* ‘OldBlush’ (**A**) and *Rosa chinensis* ‘Viridiflora’ (**B**).

**Figure 2:**
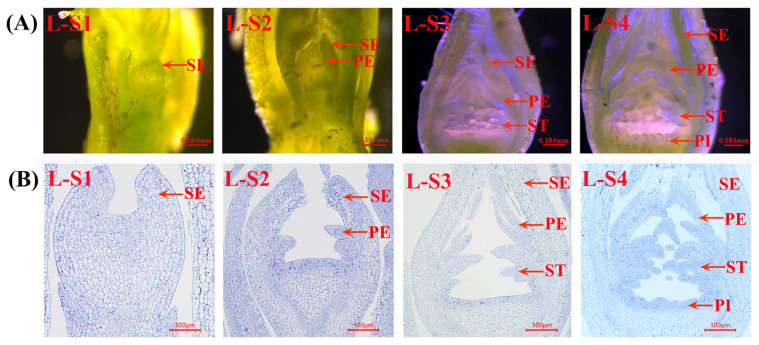
*Rosa chinensis* ‘Viridiflora’ micrograph (**A**) and semi-thin section observation (**B**). L-S1–L-S4: development stages of Viridiflora; L-S1: Sepal primordium formation period; L-S2: Petal primordium formation period; L-S3: Stamen primordium formation period; L-S4: Pistil primordium formation period; SE: sepals; PE: petals; ST: stamen; PI: pistil; the notations on the figure below are the same.

**Figure 3:**
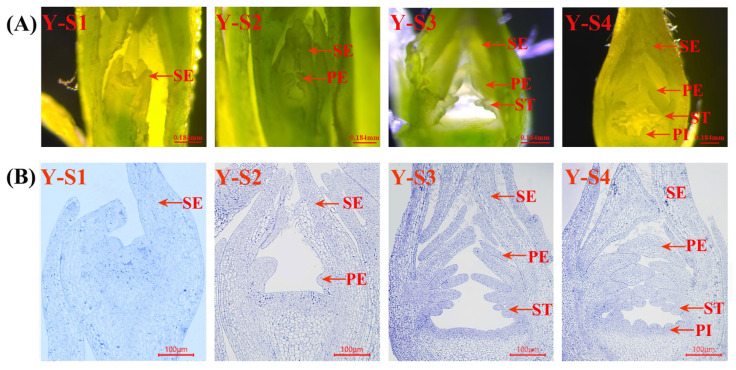
*Rosa chinensis* ‘Old Blush’ micrograph (**A**) and semi-thin section observation (**B**). Y-S1–Y-S4: development stages of Old Blush; Y-S1: Sepal primordium formation period; Y-S2: Petal primordium formation period; Y-S3: Stamen primordium formation period; Y-S4: Pistil primordium formation period; SE: sepals; PE: petals; ST: stamen; PI: pistil; the notations on the figure below are the same.

All The experimental materials consisted of flower buds from *Rosa chinensis* ‘Old Blush’ and ‘Viridiflora’ (Figure 1), with Old Blush serving as the control. Floral bud differentiation in Viridiflora and Old Blush was systematically characterized through four distinct developmental stages, designated as L-S1–L-S4 for Viridiflora (Figure 2), and Y-S1–Y-S4 for Old Blush (Figure 3). Comparative analysis revealed a highly consistent organogenesis pattern between the two cultivars, with floral organs initiating in successive whorls from the periphery toward the center of the floral meristem.

Y-S1/L-S1: Sepal Primordium Formation. The earliest morphological change involved the emergence of five triangular protrusions along the flanks of the floral apex, indicating sepal primordia initiation. These structures exhibited convex morphology with centripetal cellular expansion and typical meristematic organization.

Y-S2/L-S2: Petal Primordium Initiation. Following sepal differentiation, minute protuberances became visible adaxial to the sepal whorls, marking the onset of petal formation. Concurrently, the receptacle region expanded markedly, exhibiting upward growth and a concave basal morphology. Repeated cycles of primordia generation resulted in multilayered petal development.

Y-S3/L-S3: Stamen Primordium Differentiation. Multiple protrusions emerged proximal to the petal-receptacle junction, indicating stamen initiation. These primordia elongated progressively, eventually forming distinct staminal structures. A notable morphological divergence was observed between cultivars: Old Blush developed elliptical stamens, whereas Viridiflora exhibited elongated, taper-shaped structures.

Y-S4/L-S4: Pistil Primordium Development. The differentiation process concluded with the formation of central protrusions within the innermost floral zone, which subsequently underwent radial elongation and lateral expansion to form the pistil group. By the termination of this phase, all floral organ categories had been established, indicating complete bud differentiation.

### 2.2. Transcriptome Profiling Reveals Differential Gene Expression Pattern and Regulatory Networks During Floral Development

The quality and reliability of the transcriptomic data were assessed using total RNA from all 24 samples, which exhibited high integrity as confirmed by 1% agarose gel electrophoresis (Figure S1). CleanReads ratios ranged from 99.75% to 99.83% for Viridiflora and 99.75% to 99.86% for Old Blush, indicating high sequencing quality. Correlation analysis among three biological replicates showed intra-group coefficients of ≥0.87, demonstrating strong reproducibility and confirming the suitability of the data for further analysis. Differentially expressed genes (DEGs) between Old Blush and Viridiflora were identified using thresholds of |log_2_(FC)| > 1 and FDR < 0.05 to uncover genes potentially associated with divergent floral organ development. A total of 6535 DEGs were detected across four developmental stages, comprising 1523 up-regulated and 5012 down-regulated genes. Early stages showed limited transcriptomic divergence, with similar numbers of up- and down-regulated genes. In contrast, later stages exhibited a substantial increase in DEGs, particularly in down-regulated genes, suggesting enhanced negative regulation that may contribute to abnormal development in Viridiflora (Figure S2).

**Figure 4:**
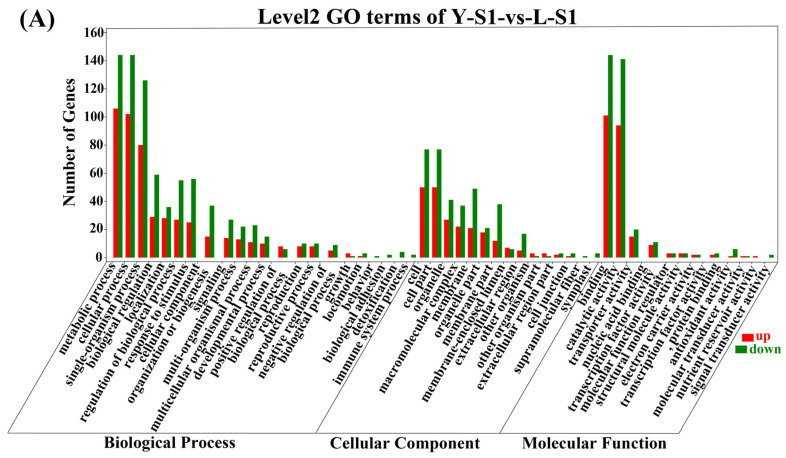

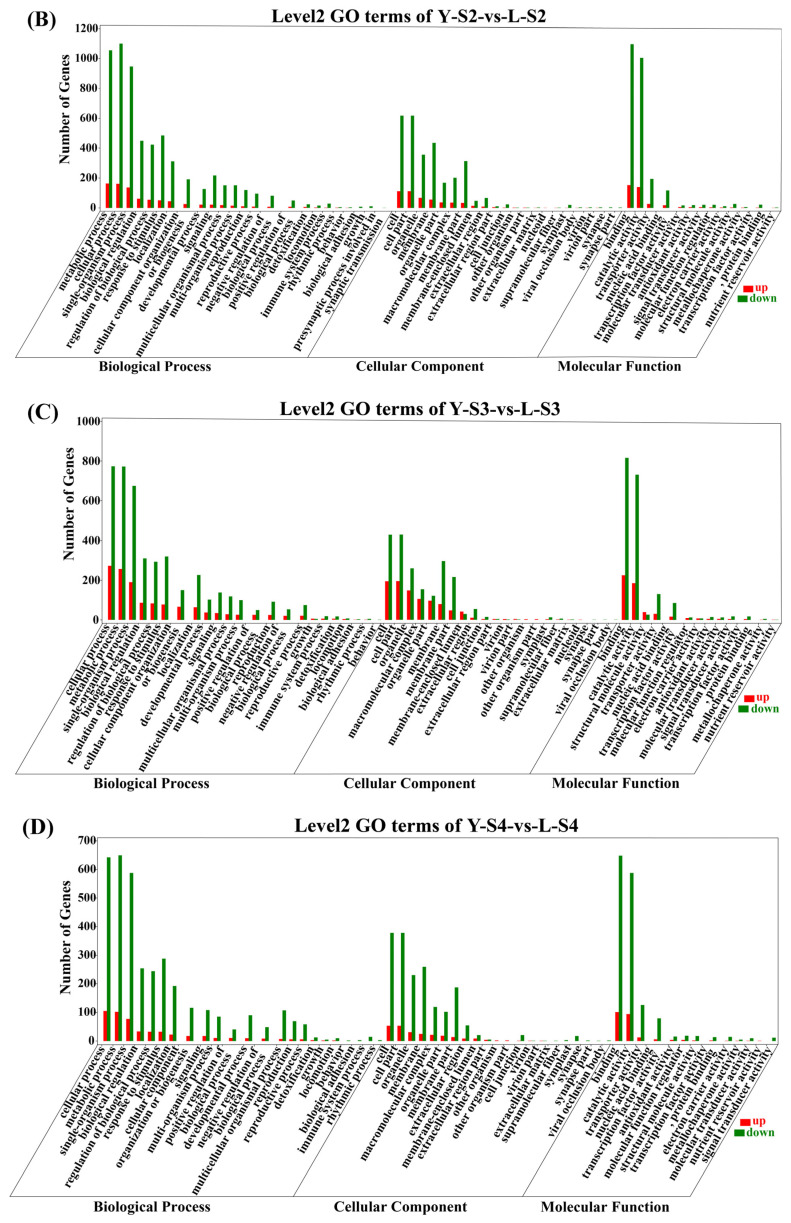
GO enrichment analysis of DEGs. (**A**) GO terms of Y-S1-vs-L-S1, (**B**) GO terms of Y-S2-vs-L-S2, (**C**) GO terms of Y-S3-vs-L-S3, (**D**) GO terms of Y-S4-vs-L-S4. Y-S1-Y-S4: development stages of Old Blush; L-S1–L-S4: development stages of Viridiflora; vs.: versus; index S1, S2, S3, and S4 in the captions represent the formation period of sepal primordium, petal primordium, stamen primordium, and pistil primordium; the notations on the figure below are the same.

**Figure 5:**
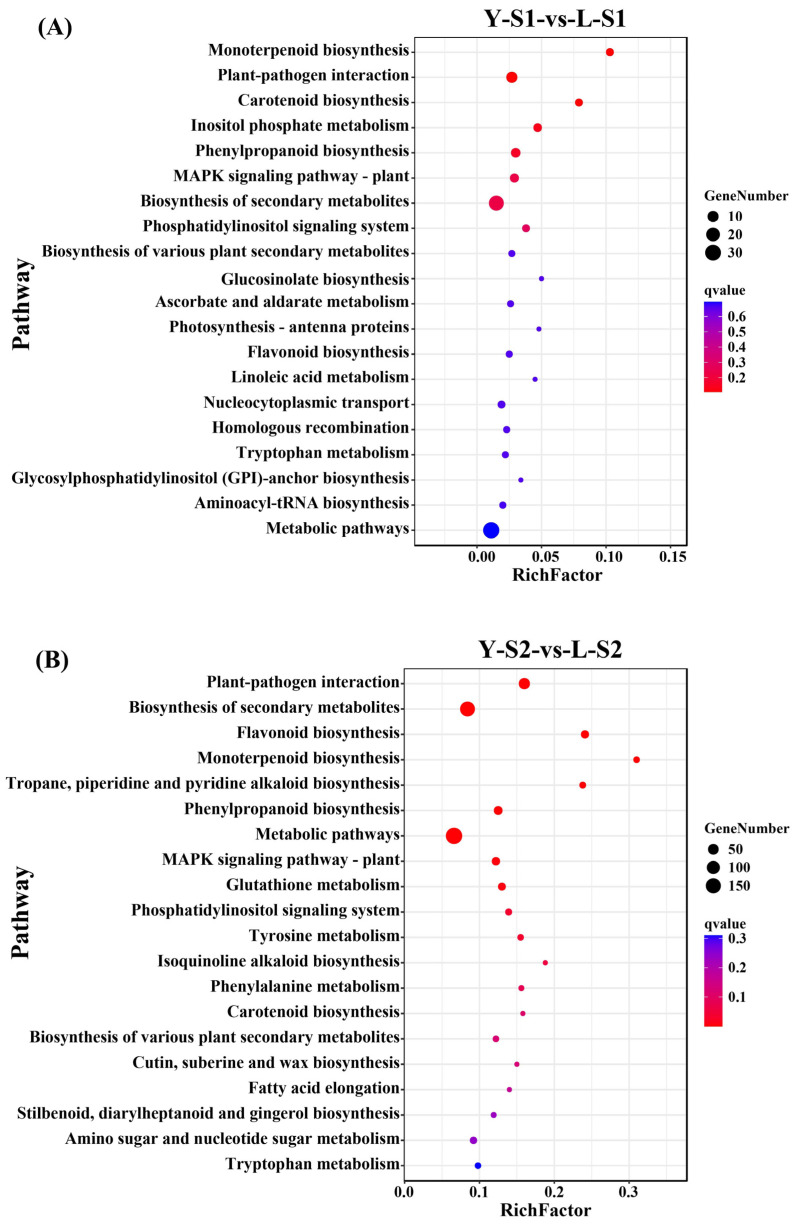

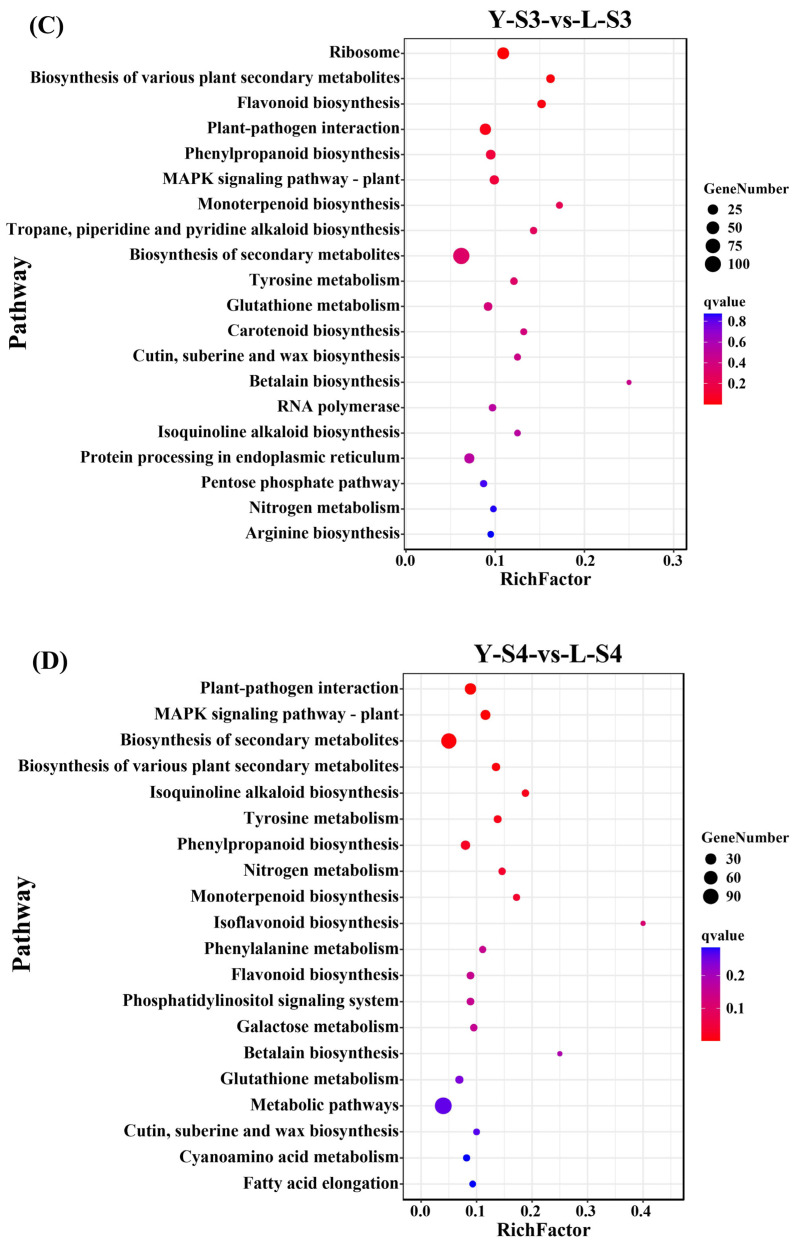
KEGG enrichment analysis of DEGs. (**A**) KEGG enrichment of Y-S1-vs-L-S1, (**B**) KEGG enrichment of Y-S2-vs-L-S2, (**C**) KEGG enrichment of Y-S3-vs-L-S3, (**D**) KEGG enrichment of Y-S4-vs-L-S4.

Functional characterization of the DEGs was carried out through GO (Gene Ontology) and KEGG (Kyoto Encyclopedia of Genes and Genomes) enrichment analyses. GO analysis revealed consistent functional profiles across stages (Figure 4), with dominant molecular functions including binding, catalytic activity, and transporter activity. Major cellular components consisted of cell, cell part, organelle, and membrane, while key biological processes involved metabolic, cellular, and single-organism processes. KEGG analysis indicated significant enrichment in secondary metabolite biosynthesis and general metabolic pathways across all stages (Figure 5), underscoring central metabolic mechanisms underlying the developmental differences.

**Figure 6:**
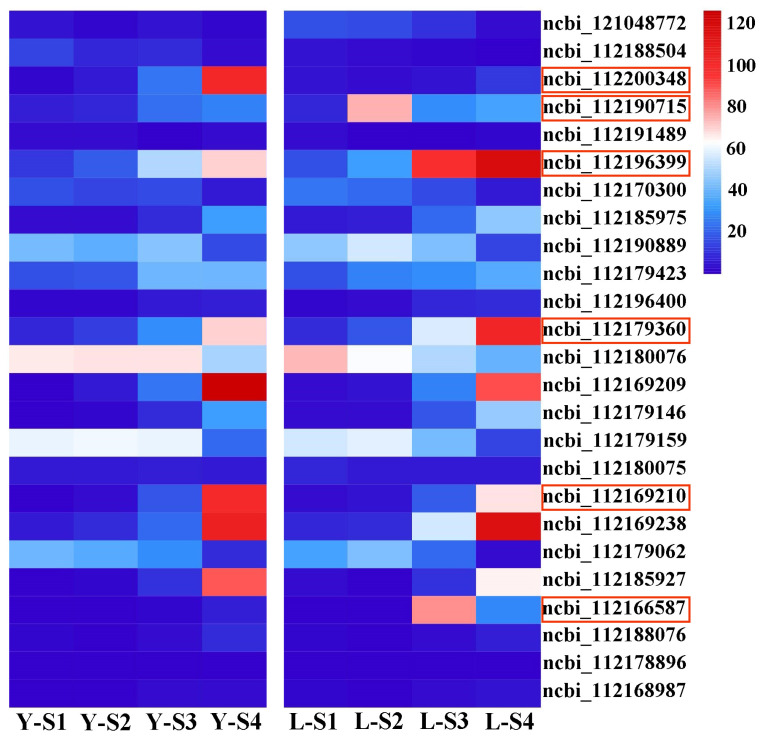
Gene expression level changes during the floral development in Old Blush (Y) and Viridiflora (L) represented by heatmap analysis. The red boxes circled in turn were 112200348 (*RcMADS4*), 112190715 (*RcFUL*), 112196399 (*RcAP1*), 112179360 (*RcMADS2*), 112169210 (*RcMADS9*), 112166587 (*RcAG2*). Y-S1/L-S1: Sepal primordium formation period; Y-S2/L-S2: Petal primordium formation period; Y-S3/L-S3: Stamen primordium formation period; Y-S4/L-S4: Pistil primordium formation period.

To pinpoint key regulatory genes, MADS-box family genes with low expression (FPKM (Fragments Per Kilobase of exon model per Million mapped fragments) < 0.5) were filtered out, and the remaining genes were analyzed via heatmap (Figure 6). Notable up-regulation in Viridiflora was observed for NCBI Gene IDs 112166587 (*RcAG2*), 112196399 (*RcAP1*), 112190715 (*RcFUL*), and 112179360 (*RcMADS2*), whereas NCBI Gene IDs 112200348 (*RcMADS4*) and 112169210 (*RcMADS9*) were significantly down-regulated. Notably, during petal primordium development, *RcFUL* expression is significantly upregulated, and this gene may influence the development of the second whorl of floral organs; during stamen primordium development, the expression level of *RcAG2* is significantly upregulated, and this gene may play a role in the abnormal development of the third whorl of floral organs. Based on functional annotation and previous studies, to explore the causes of abnormal development of petals and stamens, *RcAG2* and *RcFUL* were selected as candidate genes due to their high expression in Viridiflora. Validation using qRT-PCR on six DEGs confirmed expression trends consistent with the transcriptome data (Figure S3).

### 2.3. Phylogenetic and Protein Structural Analysis of RcAG2 and RcFUL

**Figure 7:**
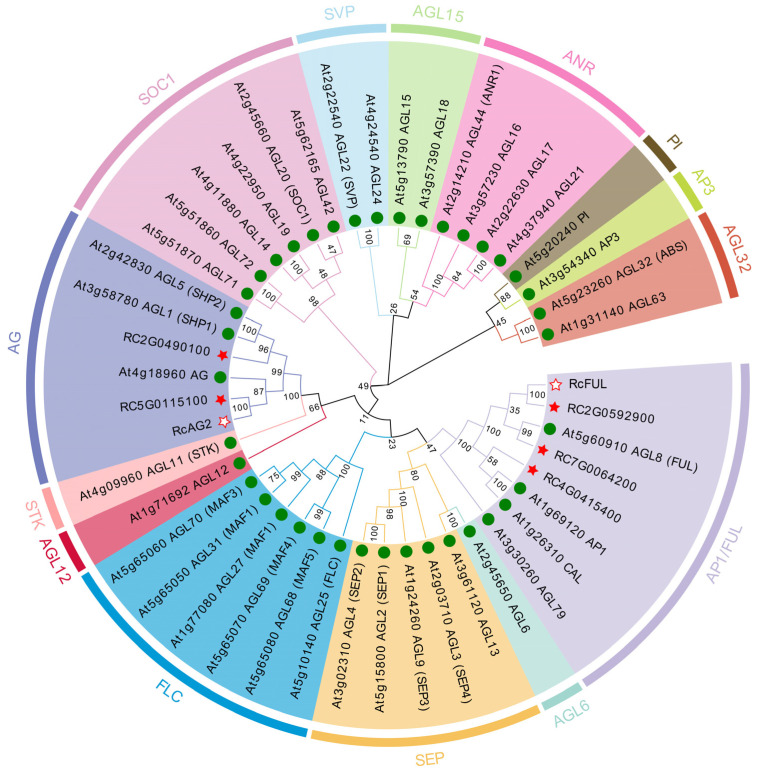
Phylogenetic tree of RcAG2 and RcFUL with the MADS-box family of *A. thaliana*. The hollow red stars were marked as genes in this study. Solid red stars represented the genes in *R. chinensis*, green circles represented the genes in *A. thaliana.*

To elucidate the evolutionary relationships of RcAG2 and RcFUL, their protein sequences were subjected to analysis against the MADS-box gene family of *A. thaliana*. The results shown that the clustering branches of RcAG2 and RcFULL differ. *RcAG2* clustered with the *AG* gene of *A. thaliana* in a single branch, categorizing it within the *AG* subfamily as a C-class gene essential for flower development. *RcFUL* and the *FUL* gene of *A. thaliana* were grouped within the same family, belonging to the *AP1/FUL* subfamily, and were classified as Class A genes involved in flower development (Figure 7).

To characterize the biochemical attributes of RcAG2 and RcFUL, their physicochemical properties were computationally predicted (Table S1). The molecular formulas were determined as C_1241_H_2006_N_380_O_388_S_8_ and C_1264_H_2065_N_375_O_395_S_11_, respectively. Both proteins exhibited theoretical isoelectric points (pI) > 7, classifying them as alkaline proteins. With instability indices exceeding 40, both were predicted to be unstable, though RcAG2 demonstrated greater stability than RcFUL. As show in Figures S4 and S5, negative grand average of hydropathicity (GRAVY) scores indicated hydrophilic characteristics for both proteins. Neither protein contained transmembrane domains or signal peptides, suggesting extracellular localization without secretory capacity. Secondary structure composition was predicted to further understand protein folding. RcAG2 consisted of 56.05% α-helices, 3.23% β-turns, 8.87% extended strands, and 31.85% random coils. RcFUL contained 51.36% α-helices, 2.33% β-turns, 8.17% extended strands, and 38.13% random coils. Homology modeling via SWISS-MODEL provided three-dimensional structural validation, while conserved domain analysis confirmed that both proteins contain K-box and MADS-MEF2-like domains, with variations only in domain arrangement.

### 2.4. Functional Characterization of RcAG2 and RcFUL

**Figure 8:**
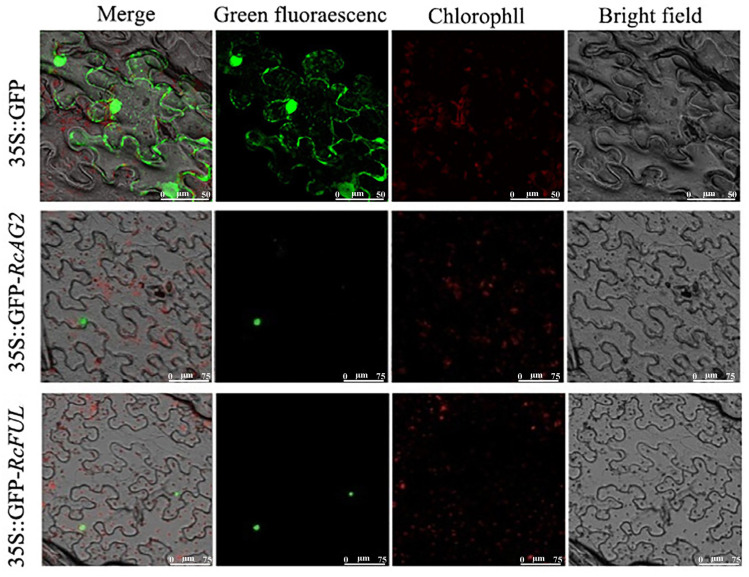
Subcellular localization of RcAG2 and RcFUL proteins. 35S::GFP: PRTL2 empty vector; 35S::GFP-*RcAG2*: Transfer vector of *RcAG2*; 35S::GFP-*RcFUL*: Transfer vector of *RcFUL*.

To determine the subcellular localization of RcAG2 and RcFUL proteins, their CDS sequences were cloned from Viridiflora flower bud cDNA. Sequencing confirmed CDS lengths of 756 bp for *RcAG2* and 774 bp for *RcFUL* (Figure S6). Recombinant plasmids were constructed and verified via colony PCR and restriction digestion, then transformed into Agrobacterium for transient expression in *Nicotiana benthamiana* leaves. Subcellular localization confirmed the nuclear localization of both RcAG2 and RcFUL, supporting their predicted roles as transcription factors (Figure 8).

**Figure 9:**
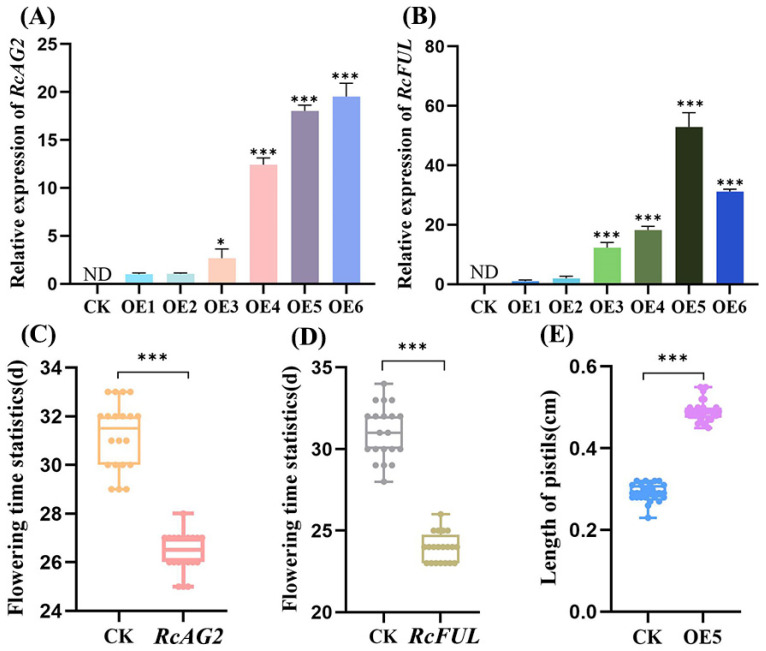
Statistical graph of expression and phenotypic data. (**A**) The expression detection map of *RcAG2* transgenic positive plants; (**B**) The expression detection map of *RcFUL* positive transgenic plants; (**C**) Statistical map of flowering time of *RcAG2* transgenic positive plants; (**D**) Statistical map of flowering time of *RcFUL* transgenic positive plants; (**E**) Pistil length statistics of positive plants OE5; OE5: Overexpression positive line 5; CK: Transgenic *Arabidopsis thaliana* plants; *: significant difference (*p* < 0.05); ***: extremely significant difference (*p* < 0.001).

To investigate the functional effects of *RcAG2* and *RcFUL*, heterologous overexpression was performed in *A. thaliana*. Positive transgenic plants were selected using antibiotic resistance and confirmed by detecting target gene expression (Figure S7). A total of 12 positive lines were successfully generated. The expression levels of *RcAG2* and *RcFUL* in transgenic lines were quantified by qRT-PCR using empty vector lines as controls. *RcAG2* expression was undetectable in controls but significantly elevated in transgenic plants, with OE4, OE5, and OE6 showing approximately 13-, 18-, and 20-fold higher expression than OE1, respectively (Figure 9A). Similarly, RcFUL expression was absent in controls but strongly induced in transgenics, with OE5 and OE6 exhibiting about 50- and 30-fold increases compared to OE1 (Figure 9B).

**Figure 10:**
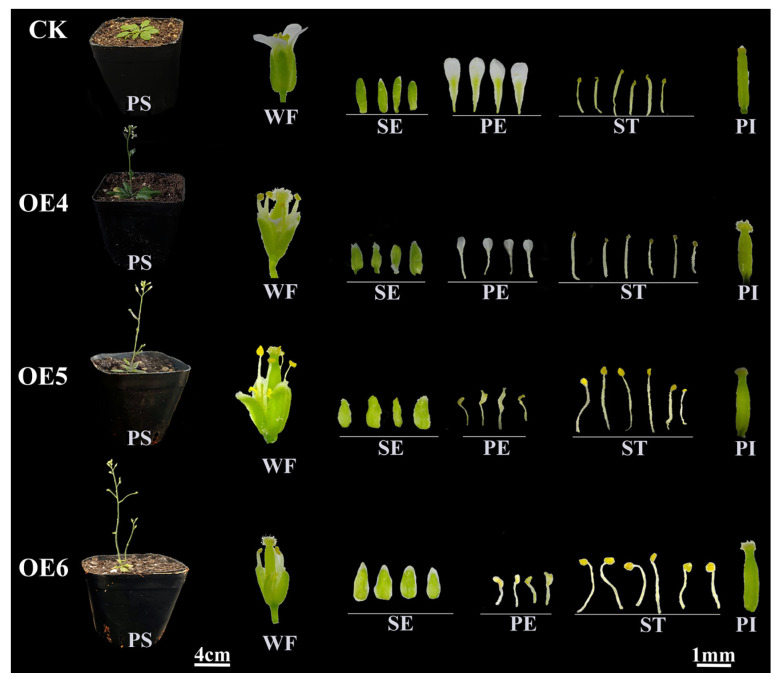
Phenotype of *RcAG2* transgenic *Arabidopsis thaliana*. OE4: Overexpression positive line 4; OE5: Overexpression positive line 5; OE6: Overexpression positive line 6; CK: Transgenic *Arabidopsis thaliana* plants; PS: Plant of Overexpression Strain; WF: Whole Flower; SE: sepals; PE: petals; ST: stamen; PI: pistil.

**Figure 11:**
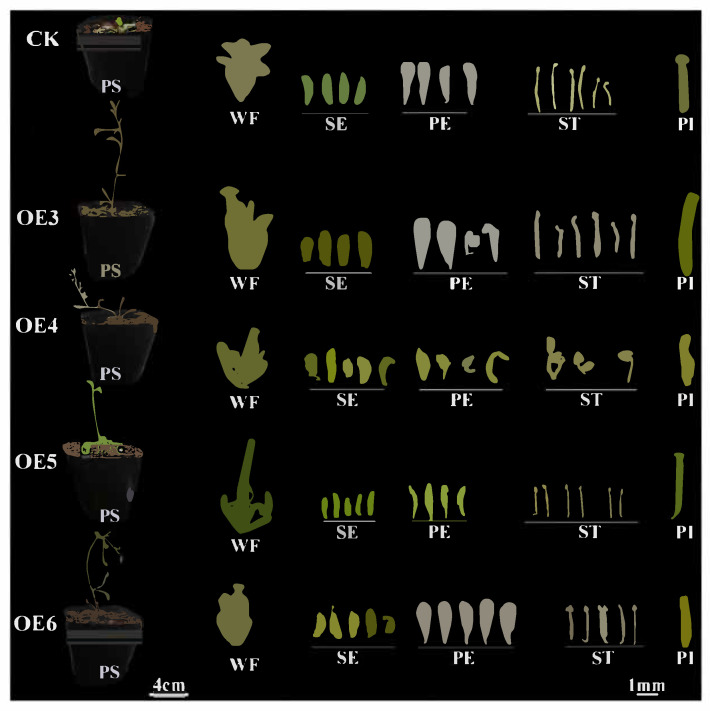
Phenotype of *RcFUL* transgenic *Arabidopsis thaliana*. OE3: Overexpression positive line 3; OE4: Overexpression positive line 4; OE5: Overexpression positive line 5; OE6: Overexpression positive line 6; CK: Transgenic *Arabidopsis thaliana* plants; PS: Plant of Overexpression Strain; WF: Whole Flower; SE: sepals; PE: petals; ST: stamen; PI: pistil.

Both *RcAG2* and *RcFUL* transgenic plants displayed early flowering phenotypes. While empty vector plants flowered around 30 days after sowing, *RcAG2* and *RcFUL* transgenics flowered approximately 4 days and 7 days earlier, respectively (Figure 9C,D). Notable alterations in floral organ morphology were observed. In *RcAG2*-overexpressing lines (OE4–OE6), sepals failed to fully enclose reproductive structures at early developmental stages, and petals exhibited abnormal morphologies such as reduced size, fan-shaped or stamen-like appearance, and light-yellow pigmentation (Figure 10 and Table S2). The inner whorl organs remained largely unaffected. *RcFUL* transgenics (OE3–OE6) also exhibited substantial floral abnormalities, including increased sepal and petal counts (up to five), greenish or leaf-like petals, reduced stamen number, and elongated pistils (~1.7-fold longer in OE5; Figure 9E and Figure 11 and Table S3). Severe phenotypic disruptions in some lines led to loss of self-pollination capacity and failure to produce seeds. Collectively, these findings indicate that heterologous expression of *RcAG2* and *RcFUL* not only promotes early flowering but also markedly disrupts floral organ development in *A. thaliana*, supporting their functional involvement in floral morphology and development.

## 3. Discussion

Floral development in roses involves a precisely timed sequence of morphological changes. The developmental process of rose flower buds can be broadly categorized into six stages: pre-differentiation, sepal primordium formation, petal primordium formation, stamen primordium formation, pistil primordium formation, and completion of floral bud differentiation [34]. Among these, the periods during which sepal, petal, stamen, and pistil primordia develop are of particular interest, as they represent critical phases for the formation of key floral organs. In alignment with these findings, the present study focused specifically on these four stages to elucidate key mechanisms underlying floral organ formation. In this study, the RNA-seq was utilized to analyze the DEGs of Old Blush and Viridiflora during the flower bud differentiation stages. The number of down-regulated genes is more than that of up-regulated genes, indicating that most genes are involved in the abnormal development of Viridiflora flowers in the form of negative regulation.

The MADS-box family is a class of important transcriptional regulatory factors related to the regulation of flower development. This study mainly screened out the DEGs of MADS-box family and obtained six genes involved in the development of floral organs: *RcAG2*, *RcAP1*, *RcFUL*, *RcMADS4*, *RcMADS9*, and *RcMADS2*. The number of DEGs is the largest during the primordial development stage of petals and stamens. The DEGs is discovered mostly during the primordial development stages of petals and stamens, and *RcAG2* and *RcFUL* were significantly expressed during these two periods were selected for research. Phylogenetic tree analysis indicates that *RcAG2* may play the role of a Class C gene, and *RcFUL* may play the role of a class A gene.

*RcAG2* gene identified as a C-class MADS-box transcription factor, plays a conserved role in floral organ development. Previous studies have shown that *AG* homologs not only regulate the differentiation and development of stamens and carpels but also regulate the termination of the floral meristem [35,36,37]. In this study, heterologous overexpression of *RcAG2* in *A. thaliana* significantly altered petal morphology, leading to reduced petal size and eventual degradation, resulting in premature exposure of stamens. Similar phenotypic changes were observed when the *AG* gene from *Prunus mume* was overexpressed in *A. thaliana* [38]. Additionally, loss of *AG* function has been reported to cause stamens to transform into petal-like structures, resulting in double flowers [39]. Although *AG* overexpression generally induces stamen petaloidy and reduces stamen number, such phenotypes were not observed in the *RcAG2*-transgenic *A. thaliana* lines in this experiment.

*RcFUL*, also part of the MADS-box family, is primarily involved in petal and sepal development. Heterologous overexpression of *RcFUL* in *A. thaliana* resulted in leaf-like petals, increased sepal number, reduced petal size, elongated stigmas, and defects in pistil and stamen development. One of the transgenic lines with low transgene expression exhibited partially normal flowers, suggesting that the constitutive 35S promoter may not fully restrict gene expression to floral organs. The use of a native promoter in future studies may help achieve more specific spatial expression. These findings are consistent with earlier reports indicating that the *FRUITFULL* (*FUL*) gene promotes inflorescence meristem formation in early development and is later expressed in carpels and siliques, also influencing leaf development [40]. Furthermore, expression of *RhFUL* in tobaccos led to smaller leaves, greenish flowers, and increased sepal number, resembling the phenotypes observed in *RcFUL*-transgenic *A. thaliana* in this study [41].

The distinct functions of *RcAG2* and *RcFUL* illustrate how divergent expression patterns and functional properties of transcription factors contribute to the complexity of floral morphology. In summary, the above results reinforce the central roles of MADS-box genes in floral development and highlight the value of rose mutants such as Viridiflora for probing the genetic regulation of floral organ identity. The functions of *RcAG2* and *RcFUL* still require further research. In the future, gene silencing technology can be utilized to directly verify their roles in roses. Searching for the upstream and downstream genes of these two genes to construct a regulatory network will enable a more in-depth analysis of the specific regulatory pathways. Meanwhile, the other genes involved in the abnormal development of Viridiflora flower organs will be further explored and identified, and the functional verification and analysis should be conducted to reveal the mechanism of rose flower organ variations.

## 4. Materials and Methods

### 4.1. Plant Materials

The all plants were cultivated in the greenhouse of the Beijing Academy of Forestry and Landscape Architecture. Flower buds at specific developmental stages, determined via microscopic observation, were collected and immediately stored at −80 °C for subsequent use. *Arabidopsis thaliana* and *Nicotiana benthamiana*, employed in later experimental steps, were maintained under controlled laboratory conditions. Healthy, disease-free Viridiflora and Old Blush plants were selected for cutting propagation. Vigorously growing branches were excised, with the basal end cut obliquely and the apical end trimmed flat, retaining 1–3 buds. Cuttings were immersed in rooting solution for approximately 3 h before being transferred into a 1:1 (*v*/*v*) mixture of vermiculite and perlite. After 60 days of rooting, the plantlets were potted, and newly developed flower buds from the following year were used for experimental analyses.

### 4.2. Morphological Analysis of Floral Bud Differentiation

Stereoscopic microscope observation: The buds of Old Blush and Viridiflora were cut in half. One-half was observed under a stereomicroscope and photographed at different stages of flower bud differentiation, while the other half was quickly stored in liquid nitrogen.

Semi-thin section method observation: Flower buds were cut from the branches of Old Blush and Viridiflora plants, then quickly put into FAA fixative and placed in a vacuum pump. The pumping process should be monitored continuously until no more bubbles appear and all the flower buds sink to the bottom of the tube; then, the vacuum was slowly released. This step was repeated 3~4 times. After the final cycle, the fixative was replaced, and samples were kept at 4 °C overnight. The fixed flower bud materials were sent to the Institute of Botany, Chinese Academy of Sciences, for subsequent operation.

### 4.3. Transcriptomic Analysis

The floral buds of Old Blush and Viridiflora at different developmental stages were collected as experimental materials, with three biological replicates per stage. Total RNA was extracted from all samples using RNAprep Pure Plant Kit (DP441) (Tiangen, Beijing, China), and transcriptomic libraries were constructed. Sequencing was performed by Beijing Qiyuan Biotechnology Co., Ltd. (Beijing, China). After quality assessment, raw reads were processed to obtain clean reads for subsequent analysis. Gene expression levels were quantified using HISAT2 [42] for alignment to the reference genome, and FPKM values were calculated. Differential gene expression analysis was conducted with DESeq2, applying thresholds of |log_2_(FC)| > 1 and FDR < 0.05 to identify significantly differentially expressed genes (DEGs). Functional enrichment analyses, including Gene Ontology (GO) and Kyoto Encyclopedia of Genes and Genomes (KEGG) pathway analyses, were performed using the Bioconductor package 3.22 “clusterProfiler 4.0” [43] to interpret the biological roles and pathways associated with the DEGs.

### 4.4. Quantitative Real-Time PCR Analysis

Gene-specific primers were designed using multiPrime [44], with *RcTCTP* [45,46] of *R. chinensis* serving as the internal reference gene (Table S4). cDNA synthesized from reverse-transcribed total RNA was used as the template. qPCR was performed using the ChamQ SYBR qPCR Master Mix kit with the following reaction system: 0.4 μL each of forward and reverse primers, 1 μL cDNA, 10 μL of 2 × ChamQ SYBR qPCR Master Mix (Sigma Aldrich, St. Louis, MO, USA), and 8.2 μL ddH_2_O, making a total reaction volume of 20 μL. The thermal cycling conditions were as follows: initial denaturation at 94 °C for 2 min; 50 cycles of denaturation at 94 °C for 5 s, annealing at 60 °C for 30 s; and a final extension at 72 °C for 10 min. Each sample was run in three technical replicates and three biological replicates. Gene expression levels were analyzed using the 2^−∆∆Ct^ method to determine relative expression changes of target genes across different developmental stages.

### 4.5. Gene Cloning and Vector Construction

Total RNA was extracted from flower buds of Viridiflora and Old Blush at various developmental stages and reverse-transcribed into cDNA. Gene-specific primers were designed using Primer 6.0 (Table S5). The target genes were amplified using Viridiflora cDNA as the template under the following PCR conditions: initial denaturation at 95 °C for 3 min; 30 cycles of denaturation at 95 °C for 15 s, annealing at 58 °C for 20 s, and extension at 72 °C for 1 min; followed by a final extension at 72 °C for 5 min. The 50 μL reaction system consisted of: ddH_2_O, 2 μL each of forward and reverse primers, 10 pg–30 ng cDNA, and 25 μL of 2× Phanta Max Master Mix (Dye Plus) (Vazyme, Nanjing, China). After confirming the correctness of the target gene sequences by sequencing, primers containing appropriate restriction sites were designed. The amplified fragments were ligated into the overexpression vector (pCAMBIA2300-35S) and the subcellular localization vector (pRTL2-GFP). The recombinant plasmids were then transformed into *Agrobacterium tumefaciens* GV3101 competent cells. Positive colonies were selected via PCR verification, and validated strains were cultured and stored at −80 °C for further use.

### 4.6. Bioinformatics Analysis

The homologous sequences of *RcAG2* and *RcFUL* genes in *A. thaliana* were identified by using the TAIR online website (https://www.arabidopsis.org/, accessed on 6 April 2025) [47], and the phylogenetic tree was constructed by using MEGA11.0 software. The physicochemical properties of RcAG2 and RcFUL proteins were analyzed by Expasy-ProtParam (https://web.expasy.org/protparam/, accessed on 6 April 2025). The secondary and tertiary structures of proteins were predicted by SOPMA and SWISS-MODEL (https://npsa.lyon.inserm.fr/cgi-bin/npsa_automat.pl?page=/NPSA/npsa_sopma.html, https://swissmodel.expasy.org/interactive, accessed on 6 April 2025). The signal peptide sites of protein sequences were predicted by SignalP 4.1 (https://services.healthtech.dtu.dk/services/SignalP-4.1/, accessed on 6 April 2025). The transmembrane regions of proteins were predicted by TMHMM 2.0 (https://services.healthtech.dtu.dk/services/TMHMM-2.0/, accessed on 6 April 2025). The protein domains of RcAG2 and RcFUL were predicted by NCBI CD-Search (https://www.ncbi.nlm.nih.gov/Structure/cdd/wrpsb.cgi, accessed on 6 April 2025); and the subcellular localization of proteins was predicted by WoLF-PSORT (https://wolfpsort.hgc.jp/, accessed on 6 April 2025).

### 4.7. Subcellular Localization

The Agrobacterium strains of the empty vector (pRTL2-GFP) and the re-engineered vector (pRTL2-*RcAG2*-GFP and pRTL2-*RcFUL*-GFP) were shaken, collected, and resuspended. The OD600 of the bacterial solution was adjusted to 0.6–1.0, and incubated in the dark at 28 °C for 3 h. The leaves of *N. benthamiana* growing for 4 weeks were selected, injected, and immersed until the whole leaves were moist, then dark cultured for 48~72 h. The lower epidermis of the leaves was torn off and made into temporary pieces. The slides were placed on a laser confocal microscope to observe GFP fluorescent.

### 4.8. Heterologous Overexpression

The wild-type Arabidopsis seeds were disinfected and seeded on 1/2 MS medium. Following vernalization, they were cultured under light at 25 °C. After two true leaves were grown, they were transplanted into the substrate and cultured at room temperature. The Agrobacterium liquid of the empty vector (pCAMBIA2300-35S) and the re-engineered vector (pCAMBIA2300-35S-*RcAG2* and pCAMBIA2300-35S-*RcFUL*) was propagated, respectively. After resuspension, the OD600 was adjusted to 0.6–0.8, and Silwet L-77 (Beijing, China) with 0.075% concentration was added for activation. *A. thaliana* was infected by inflorescence infection method (absorbed the infection solution drops onto the unflowering inflorescences and cultivated them for 1 d in the dark, then repeated the infection once after 7 d), and conventional light culture was carried out after 24 h of dark treatment. After obtaining the T1 transgenic Arabidopsis plants, the positive plants were screened, the RNA was extracted from the inflorescence, and the cDNA was obtained by reverse transcription. The specific primers with restriction sites were used for PCR identification, and the expression of the target gene in the positive Arabidopsis plants was detected by real-time fluorescence quantitative detection. *AtACT7* (F: GTATGCTCTTCCTCATGCTATCCTT; R: TTCCCGTTCTGCGGTAGTG) was used as the internal reference gene.

## 5. Conclusions

In conclusion, *RcAG2* and *RcFUL* were screened and cloned in this study. Both genes have typical MADS-box family domains. Subcellular localization showed that all of them function in the nucleus. Overexpression of *RcAG2* showed petal shrinkage like stamen phenomenon; overexpression of *RcFUL* showed sepal number increase and could not bear fruit normally. These results suggest that both genes can affect the normal development of flower organs, but the regulation of flower organs in *R. chinensis* is unknown. The results of this study laid a foundation for exploring the special flower type of green calyx and provided a certain theoretical basis for rose flower type breeding.

## Data Availability

Data are contained within the article; additional inquiries can be addressed directly to the corresponding authors.

## Supplementary Materials

The following supporting information can be downloaded at: https://www.mdpi.com/article/10.3390/plants15010011/s1, Table S1. Prediction of physicochemical properties of gene-encoded proteins. Table S2. Phenotype of *RcAG2* transgenic *Arabidopsis thaliana*. Table S3. Phenotype of *RcFUL* transgenic *Arabidopsis thaliana*. Table S4. qRT-PCR primer sequences. Table S5. Gene primer sequences. Figure S1. Electrophoresis results of total RNA insequencing samples. 1-24 correspond to 24 samples. Figure S2. Volcano plot of DEGs. (A) Y-S1-vs-L-S1 volcano plot, (B) Y-S2-vs-L-S2 volcano plot, (C) Y-S3-vs-L-S3 volcano plot, (D) Y-S4-vs-L-S4 volcano plot. Y-S1-Y-S4: development stages of Old Blush; L-S1-L-S4: development stages of Viridiflora; VS: versus; index S1, S2, S3, and S4 in the captions represent the formation period of sepal primordium, petal primordium, stamen primordium, and pistil primordium. Figure S3. Validation of differentially expressed gene level. *RcFUL* (112190715); *RcAG2* (112166587); *RcMADS3* (112179146); *RcMADS2*-1 (112185975); *RcAP2* (112187948); *RcAP2-3* (112191477). Figure S4. Summary of bioinformatics analysis of RcAG2. (A) Protein transmembrane domain prediction; (B) Protein signal peptide prediction; (C) Protein secondary structure prediction; (D) Protein tertiary structure prediction; (E) Protein conserved domain prediction. Figure S5. Summary of bioinformatics analysis of RcFUL. (A) Protein transmembrane domain prediction; (B) Protein signal peptide prediction; (C) Protein secondary structure prediction; (D) Protein tertiary structure prediction; (E) Protein conserved domain prediction. Figure S6. Cloning the CDS sequence of *RcAG2* and *RcFUL*. M: Marker 2000; 1,2: the CDS sequence of *RcAG2*; 3,4: the CDS sequence of *RcFUL*. Figure S7. Verification results of pCAMBIA2300-*RcAG2* (A) and pCAMBIA2300-*RcFUL* (B) transgenic *Arabidopsis thaliana*. M: Marker 2000; WT: Wild-type *Arabidopsis thaliana*; pCAMBIA2300: Transgenic *Arabidopsis thaliana* plants.

## References

[1] Liu J., Fu X., Dong Y., Lu J., Ren M., Zhou N., Wang C. (2018). Mikcc-type mads-box genes in *Rosa chinensis*: The remarkable expansion of abcde model genes and their roles in floral organogenesis. Hortic. Res..

[2] Chmelnitsky I., Azizbekova N., Khayat E., Zieslin N. (2002). Morphological development of normal and phyllody expressing *Rosa hybrida* cv. Motrea flowers. Plant Growth Regul..

[3] Bendahmane M., Dubois A., Raymond O., Bris M.L. (2013). Genetics and genomics of flower initiation and development in roses. J. Exp. Bot..

[4] Dubois A., Carrere S., Raymond O., Pouvreau B., Cottret L., Roccia A., Onesto J., Sakr S., Atanassova R., Baudino S. (2012). Transcriptome database resource and gene expression atlas for the rose. BMC Genom..

[5] Wang H., Zhang R.C.J., Duan X., Zhao H., Shan H., Kong H. (2019). Diversity of flowers in basic structure and its underlying molecular mechanisms. Sci. Sin. (Vitae).

[6] Yoshida K., Oyama K., Kondo T. (2021). Insight into chemical mechanisms of sepal color development and variation in hydrangea. Proc. Jpn. Acad. Ser. B.

[7] Liu S., Song W., Pan W., Wu G. (2024). Transcriptome sequencing and SSR prediction of clematis calyx based on SMRT sequencing platform. Sci. Rep..

[8] Sharma B., Kramer E.M. (2017). Aquilegia b gene homologs promote petaloidy of the sepals and maintenance of the c domain boundary. Evodevo.

[9] Dubois A., Raymond O., Maene M., Baudino S., Langlade N.B., Boltz V., Vergne P., Bendahmane M. (2010). Tinkering with the c-function: A molecular frame for the selection of double flowers in cultivated roses. PLoS ONE.

[10] Fan M., Li X., Zhang Y., Wu S., Song Z., Yin H., Liu W., Fan Z., Li J. (2022). Floral organ transcriptome in *Camellia sasanqua* provided insight into stamen petaloid. BMC Plant Biol..

[11] Fan Y., Jin X., Wang M., Liu H., Tian W., Xue Y., Wang K., Li H., Wu Y. (2024). Flower morphology, flower color, flowering and floral fragrance in *Paeonia* L. *Front*. Plant Sci..

[12] Qiu T., Li S., Zhao K., Jia D., Chen F., Ding L. (2023). Morphological characteristics and expression patterns of cmcyc2c of different flower shapes in *Chrysanthemum morifolium*. Plants.

[13] Pan Z.J., Chen Y.Y., Du J.S., Chen Y.Y., Chung M.C., Tsai W.C., Wang C.N., Chen H.H. (2014). Flower development of *Phalaenopsis* orchid involves functionally divergent sepallata-like genes. New Phytol..

[14] Kaufmann K., Wellmer F., Muiño J.M., Ferrier T., Wuest S.E., Kumar V., Serrano-Mislata A., Madueno F., Krajewski P., Meyerowitz E.M. (2010). Orchestration of floral initiation by apetala1. Science.

[15] Krizek B.A., Meyerowitz E.M. (1996). The arabidopsis homeotic genes apetala3 and pistillata are sufficient to provide the b class organ identity function. Development.

[16] Pelaz S., Tapia-López R., Alvarez-Buylla E.R., Yanofsky M.F. (2001). Conversion of leaves into petals in arabidopsis. Curr. Biol..

[17] Du C., Zhang H., Luo X., Song Y., Bi J., Wang Y., Zhang H. (2024). Progress in gene regulation of plant floral organ development. J. Plant Genet. Resour..

[18] Honma T., Goto K. (2001). Complexes of mads-box proteins are sufficient to convert leaves into floral organs. Nature.

[19] Soltis P.S., Soltis D.E., Kim S., Chanderbali A., Buzgo M. (2006). Expression of floral regulators in basal angiosperms and the origin and evolution of abc-function. Adv. Bot. Res..

[20] Theißen G., Melzer R., Rümpler F. (2016). Mads-domain transcription factors and the floral quartet model of flower development: Linking plant development and evolution. Development.

[21] Chmelnitsky I., Khayat E., Zieslin N. (2003). Involvement of rag, a rose homologue of agamous, in phyllody development *of Rosa Hybrida* cv. Motrea. Plant Growth Regul..

[22] Gao Z., Zhang M., Wang S., Zhang Z. (2008). Research progress in floral organ identity gene *agamous*. Acta Bot. Boreali-Occident. Sin..

[23] Li R., Sui M., Wang Q., Yu C., Du G., Yan H. (2021). Cloning and analysis of rcag promoter in *Rosa chinensis* ‘viridiflora’. Plant Sci. J..

[24] Galimba K.D., Tolkin T.R., Sullivan A.M., Melzer R., Theißen G., Di Stilio V.S. (2012). Loss of deeply conserved c-class floral homeotic gene function and c-and e-class protein interaction in a double-flowered ranunculid mutant. Proc. Natl. Acad. Sci. USA.

[25] Lu J., Wang W., Fan C., Sun J., Yuan G., Guo Y., Yu X., Chang Y., Liu J., Wang C. (2024). Telo boxes within the agamous second intron recruit histone 3 lysine 27 methylation to increase petal number in rose (*Rosa chinensis*) in response to low temperatures. Plant J..

[26] Litt A., Irish V.F. (2003). Duplication and diversification in the apetala1/fruitfull floral homeotic gene lineage: Implications for the evolution of floral development. Genetics.

[27] Shan H., Zhang N., Liu C., Xu G., Zhang J., Chen Z., Kong H. (2007). Patterns of gene duplication and functional diversification during the evolution of the ap1/squa subfamily of plant mads-box genes. Mol. Phylogenet. Evol..

[28] Yue L., Li E., Li S., Gao R., Bai Z., Li H., Li D., Yu R. (2025). Functional analysis of *rcful* in regulating flowering time in *Rosa chinensis* ‘old blush’. Chin. J. Biotechnol..

[29] Di Marzo M., Herrera-Ubaldo H., Caporali E., Novák O., Strnad M., Balanza V., Ezquer I., Mendes M.A., de Folter S., Colombo L. (2020). SEEDSTICK controls arabidopsis fruit size by regulating cytokinin levels and FRUITFULL. Cell Rep..

[30] José Ripoll J., Bailey L.J., Mai Q., Wu S.L., Hon C.T., Chapman E.J., Ditta G.S., Estelle M., Yanofsky M.F. (2015). Microrna regulation of fruit growth. Nat. Plants.

[31] Chen W., Zhou Y., Luo P., Cui Y. (2024). Molecular mechanism of petal doubling of flower in angiosperm. Chin. Bull. Bot..

[32] Sui M., HuiJun Y., Wang Z., Qiu X., Jian H., Wang Q., Chen M., Zhang H., Tang K. (2019). Identification of microrna associated with flower organ development in *Rosa chinensis* ‘viridiflora’. Plant Sci. J..

[33] Balanzà V., Martínez-Fernández I., Sato S., Yanofsky M.F., Kaufmann K., Angenent G.C., Bemer M., Ferrándiz C. (2018). Genetic control of meristem arrest and life span in arabidopsis by a fruitfull-apetala2 pathway. Nat. Commun..

[34] Li Y., Zhang Y., Zhang J., Guo W., Liu X., Zhang L., Wang X., Liu J., Sun J. (2024). Observation on morphology of flower bud differentiation and the effect of shading on the process of rosa. Mol. Plant Breed..

[35] Li C., Dong N., Li X., Wu S., Liu Z., Zhai J. (2020). A review of mads-box genes, the molecular regulatory genes for floral organ development in orchidaceae. Acta Hortic. Sin..

[36] Wei M., Zeng X., An Z., Hu Y., Huang X., Weiguo L. (2020). Advances in the maintenance and termination of floral meristem regulated by c-type floral organ gene *agamous* (*ag*). Biotechnol. Bull..

[37] Zhao X., Zhu Y., Xiao J., Jia R., Jiang Z., He F. (2015). Advances of *mads-box* genetic diversity and evolutionary development in plants. North. Hortic..

[38] Xiao C. (2021). Functional Analysis of *Prunus mume* Pmag and Selection of Candidated Genes for Double Flower. Master’s Thesis.

[39] Ou J., Huang X., Hu Y., Wei Z., Yang G., Chen H., He J., Ma S., Fan J., Peng H. (2023). Research progress of stamen petaloidy formation mechanism. Agric. Res. Appl..

[40] Ma N., Chen W., Fan T., Tian Y., Zhang S., Zeng D., Li Y. (2015). Low temperature-induced dna hypermethylation attenuates expression of rhag, an agamous homolog, and increases petal number in rose (*Rosa hybrida*). BMC Plant Biol..

[41] Feng Y. (2024). Cloning and Functional Verification of Rhful Gene Related to Chinese Rose Development. Master’s Thesis.

[42] Kim D., Langmead B., Salzberg S.L. (2015). Hisat: A fast spliced aligner with low memory requirements. Nat. Methods.

[43] Wu T., Hu E., Xu S., Chen M., Guo P., Dai Z., Feng T., Zhou L., Tang W., Zhan L.I. (2021). Clusterprofiler 4.0: A universal enrichment tool for interpreting omics data. Innovation.

[44] Xia H., Zhang Z., Luo C., Wei K., Li X., Mu X., Duan M., Zhu C., Jin L., He X. (2023). Multiprime: A reliable and efficient tool for targeted next-generation sequencing. Imeta.

[45] Arnaud R., David L., Tatiana T., Fabien L.C., Laurence H.O., Fabrice F. (2009). A survey of flowering genes reveals the role of gibberellins in floral control in rose. Theor. Appl. Genet..

[46] Qi Y., Yang S., Su L., Yang Y., Zhang Q., Pan H. (2024). The rc*agl61* gene affects petal number by regulating the transition between stamens and petals in *Rosa chinensis*. Acta Bot. Boreali-Occident. Sin..

[47] Parenicová L., De Folter S., Kieffer M., Horner D.S., Favalli C., Busscher J., Cook H.E., Ingram R.M., Kater M.M., Davies B. (2003). Molecular and phylogenetic analyses of the complete mads-box transcription factor family in arabidopsis: New openings to the mads world. Plant Cell.

