# Supplementary material for: The Function of RcAG2 and RcFUL in the Flower Shape Change of Rosa chinensis ‘Viridiflora’
Source: Plants (Basel). 2025 Dec 19;15(1):11. doi: 10.3390/plants15010011 (PMC12788014; doi:10.3390/plants15010011)
Supplement: Supplementary file 1 [file plants-15-00011-s001.zip › plants-4000496-supplementary.pdf]

Table S1. Prediction of physicochemical properties of gene-encoded proteins.

Protein Name	Number of Amino Acids	Molecular Weight	Isoelectric Point	Instability Index	Aliphatic Index	Average Hydropathicity
RcAG2	248	28529.19	9.40	50.17	79.92	-0.785
RcFUL	257	29188.24	9.21	61.39	77.08	-0.761

Table S2. Phenotype of *RcAG2* transgenic *Arabidopsis thaliana*.

Lines	Expression Levels of Transgene ($2^{-\Delta\Delta CT}$)	Number, Size and Characteristics				Flowering Time	Number of Leaves at Flowering	Reproductive Ability
		Sepal	Petal	Stamen	Pistil			
OE1	1.01	4 pieces, 1.0-1.7 mm, no obvious change	4 pieces, 1.6-1.8 mm, a few petals area reduced	6 pieces, 1.2-1.4 mm, no obvious change	1 pieces, 1.7-2.0 mm, no obvious change	27	9	normal fruiting
OE2	1.06	4 pieces, 1.0-1.5 mm, no obvious change	4 pieces, 1.4-1.8 mm, a few petals area reduced	6 pieces, 1.1-1.4 mm, no obvious change	1 pieces, 1.7-2.0 mm, no obvious change	27	8	normal fruiting
OE3	2.67	4 pieces, 1.0-1.7 mm, no obvious change	4 pieces, 1.4-2.0 mm, a few petals curled, shaped like stamens	6 pieces, 1.0-1.4 mm, no obvious change	1 pieces, 1.7-2.0 mm, no obvious change	27	8	normal fruiting
OE4	12.42	4 pieces, 1.0-1.2 mm, Four, 1.0-1.2mm, some sepals became smaller	4 pieces, 1.1-1.3 mm, petal area reduced, bottom became slender	6 pieces, 1.0-1.5 mm, no obvious change	1 pieces, 1.5-1.9 mm, no obvious change	26	9	normal fruiting
OE5	18.04	4 pieces, 1.0-1.2 mm, some sepals became smaller	4 pieces, 0.9-1.3 mm, petals curled, shaped like stamens, color became slightly yellow	6 pieces, 1.2-1.5 mm, no obvious change	1 pieces, 1.5-2.0 mm, no obvious change	27	7	parts fruit normally
OE6	19.53	4 pieces, 1.0-1.4 mm, some sepals became smaller	4 pieces, 0.9-1.2 mm, petals curled, shaped like stamens, color became slightly yellow	6 pieces, 1.3-1.7 mm, slightly curled	1 pieces, 1.7-2.2 mm, no obvious change	26	6	parts fruit normally

* The empty vector Arabidopsis was used as the control.

Table S3. Phenotype of *RcFUL* transgenic *Arabidopsis thaliana*.

Lines	Expression Levels of Transgene ($2^{-\Delta\Delta CT}$)	Number, Size and Characteristics				Flowe ring Time	Number of Leaves at Flowering	Reprodu ctive Ability
		Sepal	Petal	Stamen	Pistil			
OE1	1.04	4 pieces (5 for a few flowers), 1.3-1.8 mm, no obvious change	4 pieces, 1.5-2.0 mm, no obvious change	6 pieces, 1.4-2.2 mm, no obvious change	1 pieces, 1.7-2.6 mm, no obvious change	24	9	parts fruit normally
OE2	1.96	4 pieces, 1.4-1.7 mm, no obvious change	4 pieces, 1.6-2.0 mm, a few petals became light green	6 pieces, 1.3-2.2 mm, no obvious change	1 pieces, 1.7-2.5 mm, no obvious change	25	8	parts fruit normally
OE3	12.31	4 pieces, 1.1-1.6 mm, no obvious change	4 pieces, 1.4-2.3 mm, a part of petals curled	6 pieces, 1.3-2.2 mm, no obvious change	1 pieces, 2.0-3.2 mm, no obvious change	24	6	a small amount fruit normally
OE4	18.25	5 pieces, 1.0-1.5 mm, no obvious change	4 pieces, 1.2-1.9 mm,a part of petals curled, color became slightly green	5 pieces, 1.1-1.9 mm, some of the anthers became swollen	1 pieces, 2.0-2.3 mm, slightly shrink	24	5	fruitless ness
OE5	52.90	5 pieces, 1.0-1.2 mm, no obvious change	4 pieces, 1.8-2.0 mm, the color became green	6 pieces, 1.4-2.0 mm, no obvious change	1 pieces, 4.5-5.5 mm, no obvious change	23	3	fruitless ness
OE6	31.14	5 pieces, 1.1-1.4 mm, no obvious change	5 pieces, 2.0-2.2 mm, no obvious change	6 pieces, 1.6-2.0 mm, no obvious change	1 pieces, 2.0-2.3 mm, no obvious change	23	4	fruitless ness

* The empty vector Arabidopsis *thaliana* was used as the control.

Table S4. qRT-PCR primer sequences.

Gene ID	Primer Names	Primer Sequences (5′-3′)
112187948	RcAP2-F	ATGCGTAGAAGAGATGGGTA
	RcAP2-R	TGGGTTAAAAGTATGTGGAG
112190715	RcFUL-F	AGCTGAAGCGGATCGAGAAC
	RcFUL-R	TCCATGCTGGAATCGGTAGC
112191477	RcAP2-3-F	CTGACTGTCCGAATGAGAAC
	RcAP2-3-R	GAAGTTGGAGTTCATGCTGT
112185975	RcMADS2-1-F	CTGCCAAGGAACTTGAGAGCAG
	RcMADS2-1-R	TCAAGGAGGACTCTAGCTGACG
112166587	RcAG2-F	CGATACAAGAAGGCATGTGCAG
	RcAG2-R	ATTGCTCAAACCCTCAGCCATA
112179146	RcMADS3-F	GCTCAAGAGAATAGAGAACAAG
	RcMADS3-R	GGGTCATACTACCAGCACTT
Actin	RcTCTP-F	GGGTGATGATGCAGCTTT
	RcTCTP-R	TTAGCACTTGACCTCCTTCA

Table S5. Gene primer sequences.

Primer Names	Primer Sequences (5′-3′)	Usage
RcAG2-F	ATGGCCTATGAAAACAAACCCAACA	Gene cloning
RcAG2-R	CCGGTCAAACTAATTGAAGGGAAAT	
RcFUL-F	ATGGGAAGAGGCAAGGTTCAG	
RcFUL-R	GCTCATTTATTCAAATGGCGAACC	
pRTL2-RcAG2-F	<u>CTCGAG</u>ATGGCCTATGAAAAC	Subcellular localization
pRTL2-RcAG2-R	GC<u>TCTAGA</u>AACTAATTGAAGGGAA	
pRTL2-RcFUL-F	<u>CTCGAG</u>ATGGGAAGAGGCAA	
pRTL2-RcFUL-R	<u>CCCGGG</u>TTTATTCAAATGGCG	
p2300-RcAG2-F	AA<u>GGATCC</u>ATGGCCTATGAAAACAAACC	Heterologous overexpression
p2300-RcAG2-R	C<u>GTCGAC</u>AACTAATTGAAGGGAAATC	
p2300-RcFUL-F	TATA<u>CCCGGG</u>ATGGGAAGAGGCAA	
p2300-RcFUL-R	CG<u>TCTAGA</u>TTTATTCAAATGGCGAACC	

* The underline is the enzyme cutting site.

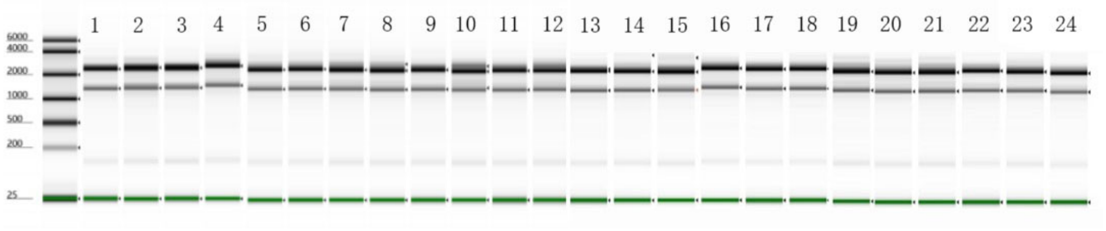

Figure S1. Electrophoresis results of total RNA insequencing samples. 1-24 correspond to 24 samples.

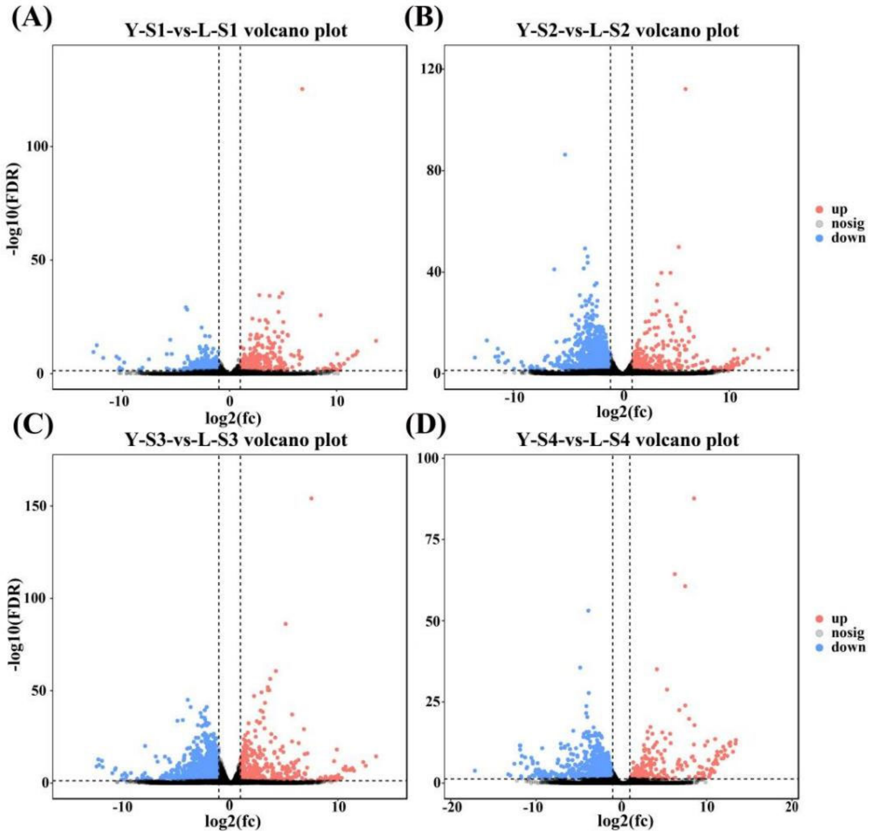

Figure S2. Volcano plot of DEGs. (A) Y-S1-vs-L-S1 volcano plot, (B) Y-S2-vs-L-S2 volcano plot, (C) Y-S3-vs-L-S3 volcano plot, (D) Y-S4-vs-L-S4 volcano plot. Y-S1-Y-S4: development stages of Old Blush; L-S1-L-S4: development stages of Viridiflora; VS: versus; index S1, S2, S3, and S4 in the captions represent the formation period of sepal primordium, petal primordium, stamen primordium, and pistil primordium.

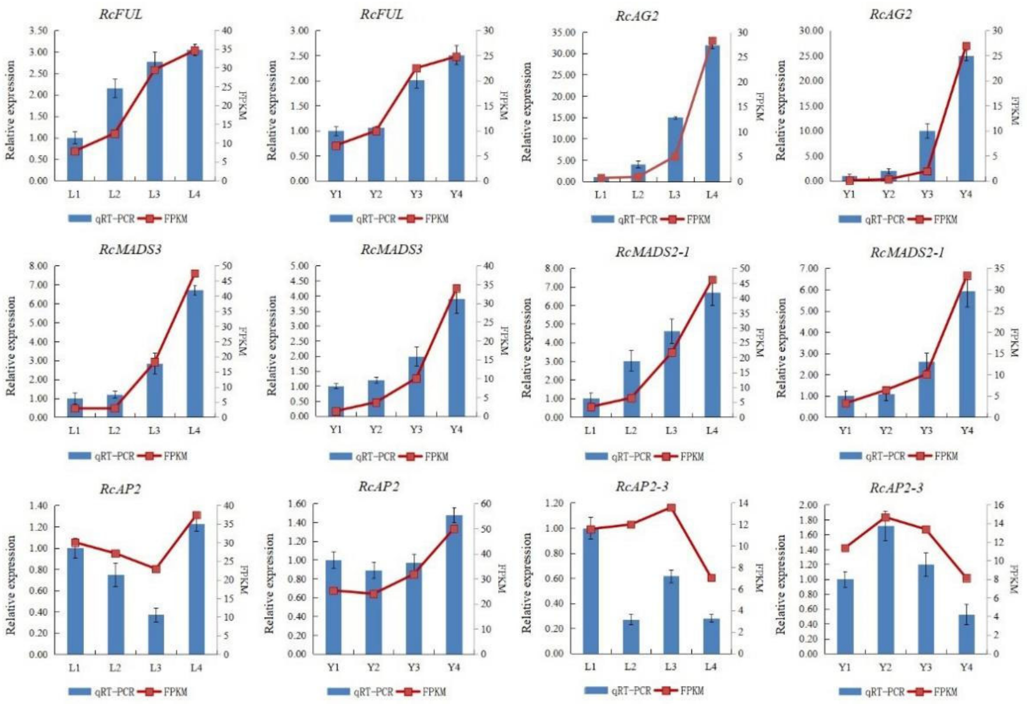

Figure S3. Validation of differentially expressed gene level. *RcFUL* (112190715); *RcAG2* (112166587); *RcMADS3* (112179146); *RcMADS2-1* (112185975); *RcAP2* (112187948); *RcAP2-3* (112191477).

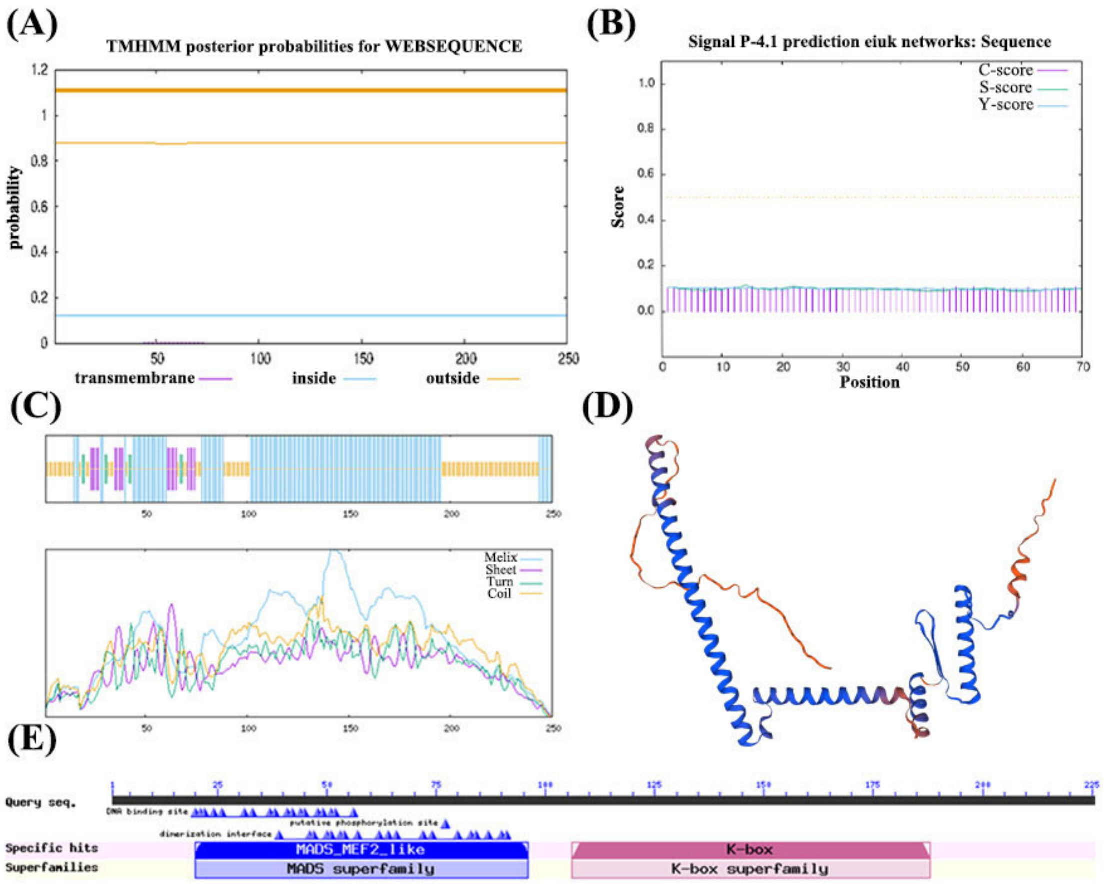

Figure S4. Summary of bioinformatics analysis of RcAG2. (A) Protein transmembrane domain prediction; (B) Protein signal peptide prediction; (C) Protein secondary structure prediction; (D) Protein tertiary structure prediction; (E) Protein conserved domain prediction.

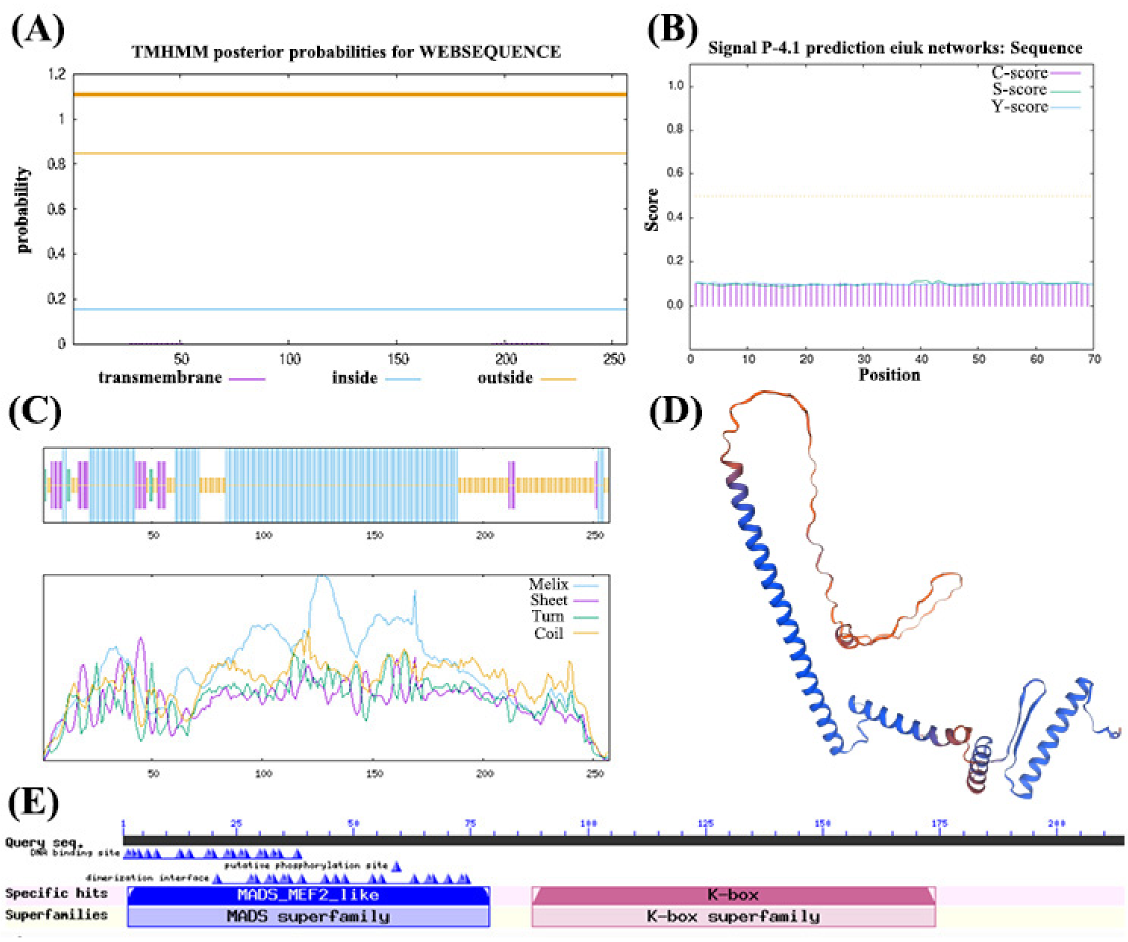

Figure S5. Summary of bioinformatics analysis of RcFUL. (A) Protein transmembrane domain prediction; (B) Protein signal peptide prediction; (C) Protein secondary structure prediction; (D) Protein tertiary structure prediction; (E) Protein conserved domain prediction.

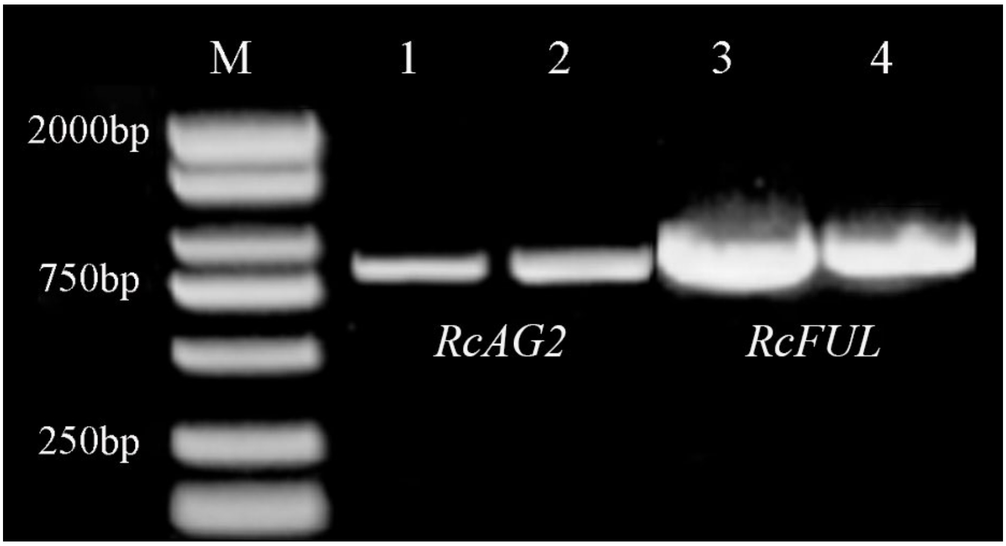

Figure S6. Cloning the CDS sequence of *RcAG2* and *RcFUL*. M: Marker 2000; 1,2: the CDS sequence of *RcAG2*; 3,4: the CDS sequence of *RcFUL*.

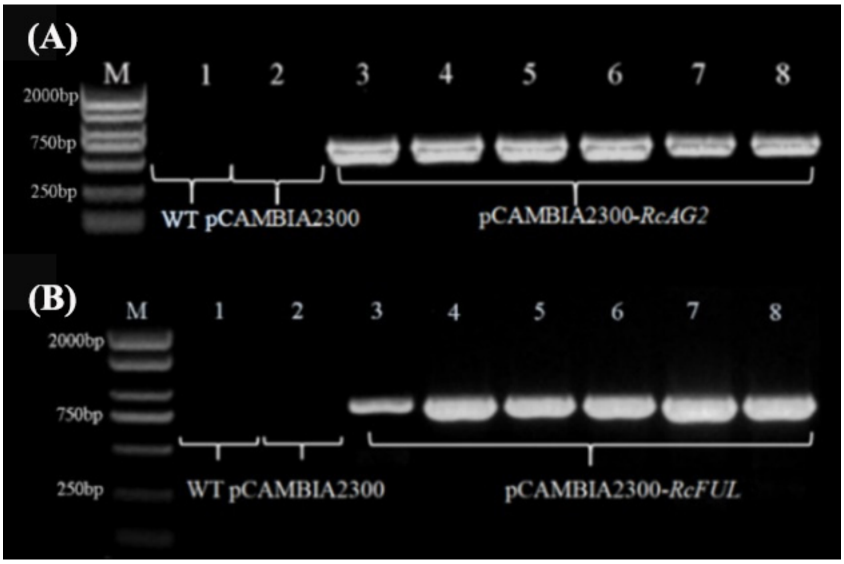

Figure S7. Verification results of pCAMBIA2300-*RcAG2* (A) and pCAMBIA2300-*RcFUL* (B) transgenic *Arabidopsis thaliana*. M: Marker 2000; WT: Wild-type *Arabidopsis thaliana*; pCAMBIA2300: Transgenic *Arabidopsis thaliana* plants.
